# Association between albumin to globulin ratio and all-cause and cardiovascular mortality among individuals with cardiovascular-kidney-metabolic syndrome: results from NHANES 2003 to 2018

**DOI:** 10.3389/fnut.2025.1622590

**Published:** 2025-07-03

**Authors:** Guangyu Wang, Guangyu Li, Pengfei Wang, Minhua Zang, Jun Pu

**Affiliations:** Department of Cardiology, School of Medicine, Renji Hospital, Shanghai Jiao Tong University, Shanghai, China

**Keywords:** albumin to globulin ratio, mortality, all-cause, cardiovascular disease, NHANES

## Abstract

**Objective:**

The albumin-to-globulin ratio (AGR) is a promising biomarker for inflammation and nutritional status. However, its association with mortality in individuals with Cardiovascular-Kidney-Metabolic (CKM) syndrome remains underexplored. This study investigates the relationship between AGR and mortality outcomes in CKM syndrome, aiming to provide insights for risk assessment and management in this population.

**Methods:**

We conducted a cohort study utilizing data from the National Health and Nutrition Examination Survey (NHANES) 2003–2018, with mortality follow-up through 31 December 2019. Survey-weighted multivariable Cox hazards regression models assessed the associations between AGR and all-cause and cardiovascular disease (CVD) mortality. Nonlinear relationships and threshold effects were evaluated using smooth curve fitting and piecewise linear regression. Sensitivity analyses, subgroup analyses, and interaction tests were further performed to validate the findings.

**Results:**

Over a median follow-up of 8.33 years, 1,745 all-cause deaths occurred, including 534 CVD-related deaths. After multivariable adjustment, a significant inverse dose–response relationship was observed between AGR and both all-cause and CVD mortality. Specifically, a nonlinear association was identified for all-cause mortality, with an inflection point at an AGR of 1.26, whereas the relationship with CVD mortality remained linear. Compared with the lowest AGR quartile, individuals in the highest AGR quartile had multivariable-adjusted hazard ratios (HR) of 0.55 (95% CI: 0.45–0.67) for all-cause mortality and 0.47 (95% CI: 0.34–0.64) for CVD mortality (P trend < 0.0001 for both outcomes). Each one-unit increase in AGR was associated with a 62% reduction in the risk of all-cause death and a 73% reduction in the risk of CVD mortality. The inverse association with all-cause mortality was more pronounced among individuals under 60 years, daily smokers, Mexican Americans and those in CKM stage 1, while the association with CVD mortality was stronger among individuals with a college degree, those under 60 years, and daily smokers. Sensitivity analyses confirmed these findings.

**Conclusion:**

Higher AGR is significantly associated with reduced risks of both all-cause and CVD mortality in the CKM syndrome population. Notably, while the relationship with all-cause mortality is nonlinear, the association with CVD mortality follows a linear pattern. These findings suggest that AGR could serve as a valuable biomarker for mortality risk stratification in CKM syndrome.

## Introduction

Cardiovascular disease (CVD), chronic kidney disease (CKD), and metabolic disorders are no longer isolated health concerns; they are converging into a fast-growing global health emergency (1–6). These conditions not only share common risk factors but also interact through complex, overlapping pathophysiological mechanisms that drive premature mortality and disability (6–8). Recognizing this interconnected burden, the American Heart Association has introduced the concept of Cardiovascular-Kidney-Metabolic (CKM) syndrome, highlighting its profound impact on both individuals and healthcare systems worldwide (7).

The scale of this crisis is staggering. In the United States, nearly one-third of adults have at least one CKM-related condition, and a growing proportion suffer from multiple overlapping diseases (9). In China, CVD alone accounts for nearly half of all deaths—underscoring the urgent need for early identification and proactive management (10). Despite the alarming mortality burden, clinical tools remain limited in their ability to detect individuals at highest risk across the CKM spectrum.

Inflammation and malnutrition are emerging as key drivers of CKM pathogenesis and outcomes (11–16). Several biomarkers—such as the systemic inflammatory response index (SIRI) (17), triglyceride glucose index (TyG) (18), neutrophil to high-density lipoprotein cholesterol (NHR) (19)—have been associated with increased mortality. Among these, the serum albumin-to-globulin ratio (AGR) is especially promising, reflecting both inflammation and nutritional status in a single, routinely available metric. While AGR has been studied in relation to CVD (20, 21), CKD (22), diabetes (23), and cancer (24), no research has yet explored its prognostic value in patients with CKM syndrome—leaving a critical gap in our understanding.

In this study, we aim to bridge that gap by leveraging data from a nationally representative cohort from the National Health and Nutrition Examination Survey (NHANES) cohort (2003–2018) to assess the association between AGR and all-cause and CVD mortality outcomes. By identifying AGR as a potential integrative biomarker for CKM risk, our study offers a simple and scalable tool for improving risk stratification—enabling earlier, more targeted interventions that meaningfully reduce the burden of this growing global syndemic.

## Participants and methods

### Study design and population

**Figure 1:**
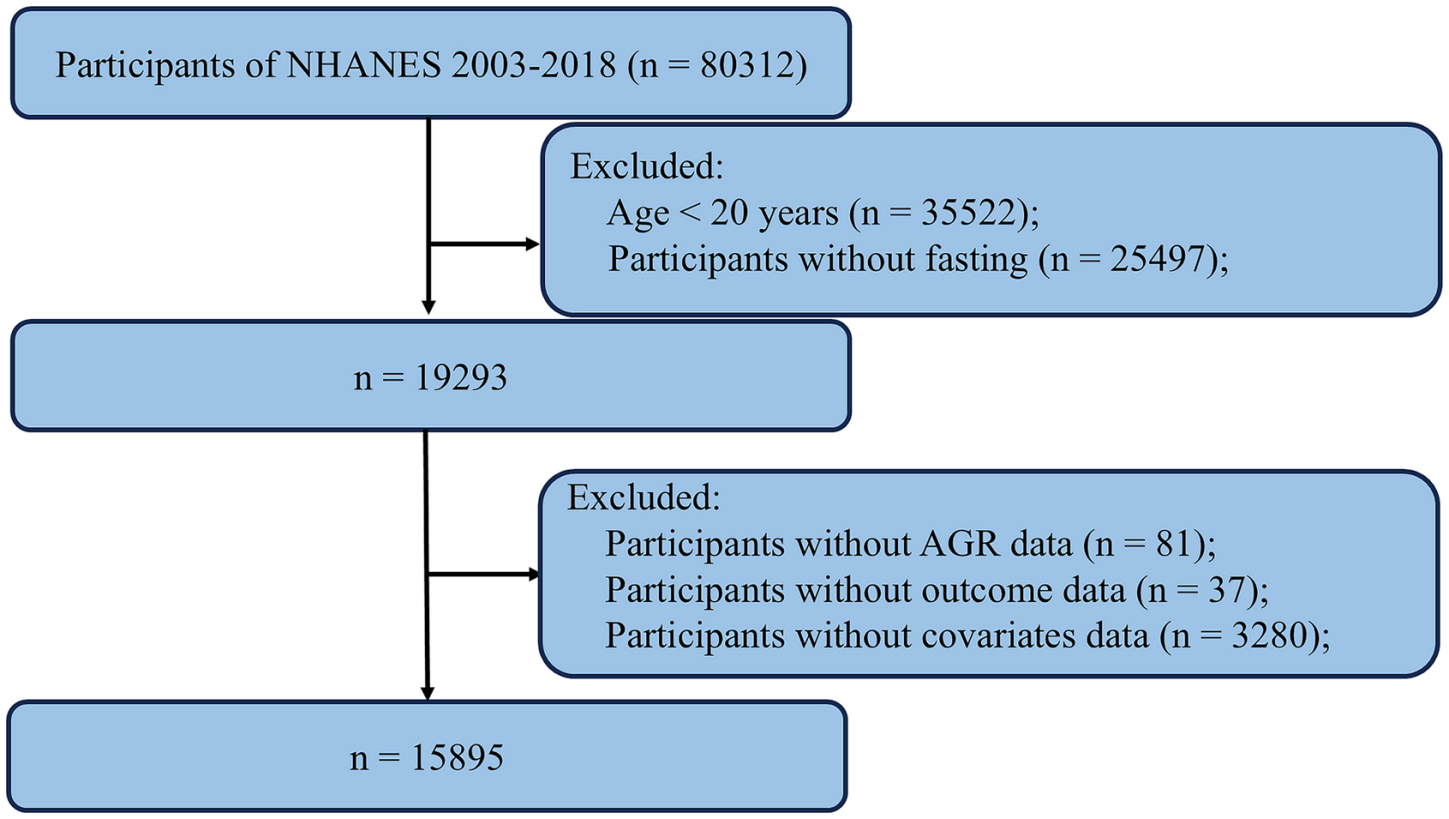
Flowchart of the sample selection from the 2003–2018 NHANES.

The data used in this study were derived from the NHANES, a publicly available national survey administered by the National Center for Health Statistics (NCHS). NHANES employs a stratified, multistage probability sampling design to select participants from the general U. S. population, ensuring representation of the civilian, noninstitutionalized population. The survey includes detailed interviews on demographic, socioeconomic, dietary, and health-related factors and is widely used in epidemiological and clinical research. All data are accessible via the NHANES website,^1^ which also provides comprehensive documentation on study design, methods, and population characteristics. The NHANES protocol was approved by the NCHS Research Ethics Review Board, and written informed consent was obtained from all participants. For this study, we compiled data from eight NHANES cycles (2003–2004 through 2017–2018). The inclusion and exclusion criteria are illustrated in the study flowchart. We first excluded 35,522 participants who were younger than 20 years. An additional 25,497 participants were excluded due to non-fasting status. We then excluded 81 participants without AGR data, 37 participants without outcome data, and 3,280 participants with missing covariate data. After applying these criteria, a total of 15,895 participants were included in the final analysis (Figure 1).

### Measurement of AGR

Albumin concentration was measured using the DxC800 system, which employs a two-color digital endpoint method. In this assay, albumin binds to bromocresol purple to form a colored complex, and the system monitors the change in absorbance at 600 nm. The change in absorbance is directly proportional to the albumin concentration in the sample. The calculation formula is as follows: AGR = albumin (g/L)/globulin (g/L).

### Assessment of covariates

Covariates were collected through standardized interviews, physical examinations, laboratory tests, and structured questionnaires administered by well-trained medical personnel. These covariates included demographic characteristics [age, sex, race/ethnicity, education levels, and ratio of family income to poverty (PIR)], medical history (hypertension and diabetes mellitus), lifestyle factors [smoking status, alcohol intake, and body mass index (BMI)] and laboratory parameters [leukocyte count, uric acid (UA), blood urea nitrogen (BUN), albumin, globulin, low-density lipoprotein cholesterol (LDL-C), high-density lipoprotein cholesterol (HDL-C) and total cholesterol (TC), triglyceride (TG), alanine aminotransferase (ALT), aspartate aminotransferase (AST), fasting blood glucose (FBG), and glycohemoglobin (HbA1c)]. In addition, the estimated glomerular filtration rate (eGFR) was calculated using the Chronic Kidney Disease Epidemiology Collaborative (CKD-EPI) equation to evaluate kidney function (25). Race/ethnicity was classified into four groups: Mexican American, non-Hispanic White, non-Hispanic Black and other race. Marital status was classified into three groups: married/living with partner, widowed/divorced/separated, and never married. Educational level was categorized into five groups: <9th grade, 9–11th grade, high school, college and graduate or above. Alcohol use was defined consuming at least 4 drinks/day. Smoking status was categorized as every day, some days, or not at all. BMI was calculated as the weight in kilograms divided by the square of height in meters, which was obtained from the body measurements. BMI was categorized as < 25, 25–29.9, and ≥ 30 kg/m^2^. PIR was categorized as < 1.3, 1.3–3.0, and ≥ 3.0. Self-reported personal interview data provided the medical and medication history of hypertension and diabetes mellitus.

During interviews, participants were asked standardized questions regarding CVD history, including: “Has a doctor or health professional ever told you that you have: congestive heart failure (CHF)/coronary heart disease (CHD)/angina/angina pectoris (AP)/heart attack/stroke?” (this question was asked separately for each condition). Participant who answered “yes” to any of these questions were classified as having CVD. Furthermore, participants were also asked whether they were currently taking prescribed medication for hypertension, diabetes, or hyperlipidemia. Those who answered “yes” were considered users of anti-hypotensive, hypoglycemic, or lipid-lowing agents, respectively.

### Assessments of CKM syndrome

CKM syndrome is defined as the coexistence of subclinical or clinical CVD, CKD, and metabolic disorders (detailed definitions are provided in Supplementary Table S1) (7). Briefly, clinical CVD includes any of the following conditions: CHF, CHD, AP, heart attack, or stroke. Subclinical CVD is defined as a ≥ 20% of 10-year CVD risk or the presence of high-risk CKD. The 10-year CVD risk was estimated using the PREVENT equations, which incorporate age, sex, tobacco use, blood pressure, cholesterol levels, diabetes status, kidney function, and the use of antihypertensive or statin therapy (see in Supplementary Table S2) (26). CKD risk was stratified based on eGFR and urinary albumin/creatinine ratio, with moderate-or high-risk classifications used for analysis (27). Metabolic disorders were defined as the presence of any of the following: overweight/obesity, abdominal obesity, prediabetes, diabetes, hypertension, dyslipidemia, and metabolic syndrome.

According to the AHA scientific statement, CKM syndrome is classified into five stage (0–4) (7). Stage 0: no detectable abnormalities. Stage 1: presence of obesity or prediabetes alone. Stage 2: obesity or prediabetes plus at least one additional metabolic disorder or CKD. Stage 3: subclinical CVD coexisting with metabolic disorders or CKD. Stage 4: clinical CVD coexisting with metabolic disorders or CKD. The full staging criteria are presented in Supplementary Table S3.

### Ascertainment of mortality

To determine mortality status during follow-up, we utilized the publicly available NHANES-linked mortality file, updated through December 31, 2019. This file was linked to the National Death Index (NDI) by the NCHS using a probabilistic matching algorithm. Follow-up time was calculated from the date of the participant interview until the date of death or the end of the mortality follow-up period (December 31, 2019), whichever occurred first. Additionally, we used the International Statistical Classification of Diseases, 10th Revision (ICD-10) to identify disease-specific deaths, with the NCHS classifying heart diseases (054–064), malignant neoplasms (019–043), and all other causes (010) for our study.

### Statistical analysis

To account for the complex sampling design of NHANES, all analyses incorporated sample weights (WTSAF2YR), clustering (SDMVPSU), and stratification (SDMVSTRA). Sample weights were calculated by dividing the 2-year fasting subsample MEC weight by 8. Weighted means ± standard error (SE) were used for continuous variables, while frequencies and weighted percentages were used for categorical variables. Multivariable Cox proportional hazards regression models were employed to evaluate the association between AGR and mortality outcomes. Covariates were added individually, and those that altered the AGR-mortality by more than 10% or were significantly associated with mortality (*p* < 0.1) were included as potential confounders in the final models. Covariates selected *a priori* based on established associations included age, sex, race, education level, marital status, diabetes mellitus, hypertension, FBG, and HbA1c. The associations of each covariate with the outcomes of interest are presented in Supplementary Tables S4–S7. Model 1 was unadjusted; Model 2 was adjusted for age, sex and race. Model 3 was adjusted for age, sex, race, education level, marital status, diabetes mellitus, hypertension, FBG, and HbA1c. To explore potential nonlinear relationships between AGR and mortality, we applied Cox hazards regression models with smooth curve fitting. Where nonlinearity was detected, threshold effect analyses were performed using two-piecewise linear regression models. Subgroup analyses were conducted by stratifying participants by age (< 60 years old or ≥ 60 years old), sex, race, education level, BMI (< 25, 25–29.9, and ≥ 30 kg/m^2^), history of diabetes mellitus or hypertension, smoking status, alcohol intake, PIR (< 1.3, 1.3–3.0, and ≥ 3.0), and CKM stage. All subgroup models were adjusted made for the covariates in model 3. Interaction terms were included to assess heterogeneity across subgroups. We also conducted several sensitivity analyses to test the robustness of our findings. First, to reduce potential reverse causality, we excluded participants who died within the first 2 years of follow-up. Second, participants with a baseline diagnosis of cancer or CVD were excluded to minimize confounding by severe illness. Third, we removed individuals using hypoglycemic, anti-hypotensive, or lipid-lowing agents at baseline to reduce potential treatment-related biases. Lastly, analyses were restricted to individuals in CKM stages 3–4. Statistical significance was defined as *p* < 0.05. Statistical analyses were performed using Empower software (X&Y solutions, Inc., Boston MA)^2^ and R version 4.2.0 (The R Foundation).^3^ This study is reported in accordance with the Strengthening the Reporting of Observational Studies in Epidemiology (STROBE) guideline (see Supplementary Table S8 for the Checklist). Additionally, a comparison between excluded and included participants is provided in Supplementary Table S9.

## Results

### Baseline characteristics

Table 1 summarizes the weighted baseline characteristics of the 15,895 participants included in this study. The mean age was 46.72 ± 0.25 years, and 48.92% were male. The weighted mean AGR was 1.52 ± 0.01, with quartiles ranges of 0.40–1.26 (Q1), 1.27–1.44 (Q2), 1.45–1.63 (Q3) and 1.64–5.88 (Q4). Significant differences across AGR quartiles were observed for variables including age, sex, race, education level, marital status, PIR, BMI, smoker status, diabetes mellitus, hypertension, leukocyte, TG, TC, HDL-C, LDL-C, FBG, HbA1c, ALT, albumin, globulin, BUN, eGFR, use of antihypertensive or hypoglycemic agents, CKM stage, all-cause mortality and CVD mortality (all *p* < 0.05). No significant differences were found for alcohol use, AST, UA, or use of lipid-lowing agents (all *p* > 0.05).

**Table 1:** Weighted baseline characteristics of participants with CKM syndrome according to AGR in NHANES 2003–2018.

Characteristics	AGR quarters	Overall	Q1 (0.40–1.26)	Q2 (1.27–1.44)	Q3 (1.45–1.63)	Q4 (1.64–5.88)	*p*-value
	*n* = 15,895	*n* = 3,959	*n* = 3,939	*n* = 4,023	*n* = 3,974
Age (year)	46.72 ± 0.25	47.54 ± 0.43	47.29 ± 0.39	46.80 ± 0.39	45.79 ± 0.40	0.0103
Sex, % (SE)						< 0.0001
Male	48.92 (0.47)	32.33 (1.11)	40.35 (1.06)	53.10 (1.19)	60.93 (0.89)	
Female	51.08 (0.47)	67.67 (1.11)	59.65 (1.06)	46.90 (1.19)	39.07 (0.89)	
Race, % (SE)						< 0.0001
Mexican American	8.50 (0.65)	12.62 (1.04)	10.17 (0.85)	8.78 (0.76)	4.75 (0.51)	
Non-Hispanic White	68.06 (1.20)	47.54 (1.85)	62.53 (1.46)	70.16 (1.44)	81.89 (0.93)	
Non-Hispanic Black	10.75 (0.65)	23.48 (1.35)	13.56 (0.84)	8.06 (0.65)	3.77 (0.39)	
Other Race	12.69 (0.60)	16.37 (0.97)	13.74 (0.81)	13.01 (0.91)	9.59 (0.56)	
Education level, % (SE)						< 0.0001
Less than 9th grade	5.49 (0.29)	8.05 (0.54)	6.99 (0.48)	5.00 (0.45)	3.37 (0.36)	
9-11th grade	10.49 (0.46)	13.52 (0.67)	11.43 (0.70)	10.28 (0.64)	8.29 (0.69)	
High school graduate	23.17 (0.58)	26.47 (1.08)	22.67 (1.03)	23.35 (1.04)	21.49 (0.88)	
College degree	31.40 (0.67)	31.36 (1.22)	31.78 (1.06)	32.45 (1.16)	30.28 (1.04)	
College and above	29.44 (0.94)	20.57 (0.97)	27.11 (1.23)	28.91 (1.25)	36.57 (1.42)	
Marital status, % (SE)						< 0.0001
Married/ Living with partner	65.09 (0.66)	59.92 (1.09)	63.07 (1.28)	67.10 (0.99)	67.78 (1.02)	
Widowed/divorced/separated	17.18 (0.43)	21.48 (0.82)	19.70 (0.84)	16.85 (0.69)	13.23 (0.66)	
Never married	17.73 (0.60)	18.59 (0.89)	17.23 (0.96)	16.06 (0.85)	18.99 (0.91)	
PIR, % (SE)						< 0.0001
< 1.3	20.30 (0.69)	28.37 (1.14)	22.41 (1.00)	19.01 (0.91)	15.42 (0.74)	
1.3–3.0	29.25 (0.70)	32.68 (1.08)	31.28 (1.12)	28.90 (1.10)	26.24 (1.02)	
≥ 3.0	50.45 (1.00)	38.94 (1.45)	46.31 (1.44)	52.09 (1.46)	58.34 (1.18)	
Smoking status, % (SE)						0.0003
Every day	20.38 (0.58)	18.84 (0.96)	19.04 (0.80)	21.39 (0.93)	21.37 (1.04)	
Some day	24.99 (0.60)	23.50 (0.95)	23.33 (1.06)	25.03 (0.95)	26.99 (0.99)	
Not at all	54.63 (0.71)	57.66 (1.32)	57.63 (1.12)	53.58 (1.02)	51.64 (1.19)	
Diabetes mellitus, % (SE)						< 0.0001
Yes	8.85 (0.30)	13.69 (0.72)	10.83 (0.70)	7.36 (0.55)	5.94 (0.47)	
No	91.15 (0.30)	86.31 (0.72)	89.17 (0.70)	92.64 (0.55)	94.06 (0.47)	
Hypertension, % (SE)						< 0.0001
Yes	30.80 (0.62)	36.55 (1.21)	32.13 (1.15)	30.54 (1.10)	26.80 (0.99)	
No	69.20 (0.62)	63.45 (1.21)	67.87 (1.15)	69.46 (1.10)	73.20 (0.99)	
Alcohol user, % (SE)						0.5483
Yes	15.77 (0.49)	15.52 (1.20)	15.03 (0.82)	16.75 (0.85)	15.58 (0.83)	
No	84.23 (0.49)	84.48 (1.20)	84.97 (0.82)	83.25 (0.85)	84.42 (0.83)	
BMI (kg/m^2^)	28.76 ± 0.09	31.61 ± 0.17	29.49 ± 0.17	28.29 ± 0.13	27.02 ± 0.14	< 0.0001
Laboratory parameters						
Leukocyte (1,000 cell/μL)	6.79 ± 0.03	7.37 ± 0.06	6.88 ± 0.04	6.68 ± 0.05	6.48 ± 0.05	< 0.0001
TG (mmol/L)	1.43 ± 0.01	1.48 ± 0.03	1.46 ± 0.02	1.45 ± 0.02	1.36 ± 0.02	0.0008
TC (mmol/L)	5.00 ± 0.01	5.03 ± 0.03	5.05 ± 0.02	5.07 ± 0.02	4.91 ± 0.02	< 0.0001
HDL-C (mmol/L)	1.41 ± 0.01	1.39 ± 0.01	1.40 ± 0.01	1.39 ± 0.01	1.44 ± 0.01	0.0012
LDL-C (mmol/L)	2.95 ± 0.01	2.97 ± 0.02	2.98 ± 0.02	3.02 ± 0.02	2.87 ± 0.02	< 0.0001
FBG (mg/dL)	104.94 ± 0.34	110.03 ± 0.70	107.38 ± 0.83	103.40 ± 0.52	101.63 ± 0.42	< 0.0001
HbA1c (%)	5.58 ± 0.01	5.85 ± 0.02	5.67 ± 0.02	5.52 ± 0.02	5.41 ± 0.01	< 0.0001
ALT (U/L)	24.09 ± 0.12	22.82 ± 0.33	23.67 ± 0.29	24.99 ± 0.27	24.36 ± 0.21	< 0.0001
AST (U/L)	24.15 ± 0.11	24.03 ± 0.37	23.78 ± 0.19	24.37 ± 0.18	24.31 ± 0.17	0.0846
Albumin (g/dL)	4.23 ± 0.01	3.87 ± 0.01	4.13 ± 0.01	4.28 ± 0.01	4.47 ± 0.01	< 0.0001
Globulin (g/dL)	2.87 ± 0.01	3.47 ± 0.01	3.05 ± 0.01	2.79 ± 0.01	2.45 ± 0.01	< 0.0001
Uric acid (mg/dL)	5.44 ± 0.02	5.45 ± 0.03	5.38 ± 0.03	5.48 ± 0.03	5.46 ± 0.03	0.0916
Blood urea nitrogen (mg/dL)	13.26 ± 0.07	12.70 ± 0.13	13.01 ± 0.15	13.32 ± 0.11	13.69 ± 0.10	< 0.0001
eGFR (ml/min/1.73m^2^)	98.56 ± 0.34	100.62 ± 0.62	98.63 ± 0.51	98.19 ± 0.46	97.65 ± 0.48	0.0006
Drug use						
Hypoglycemic agent, % (SE)						< 0.0001
Yes	7.05 (0.28)	10.21 (0.60)	9.07 (0.68)	5.66 (0.49)	5.00 (0.42)	
No	92.95 (0.28)	89.79 (0.60)	90.93 (0.68)	94.34 (0.49)	95.00 (0.42)	
Hypotensive agent, % (SE)						< 0.0001
Yes	22.43 (0.56)	26.98 (1.00)	23.65 (1.01)	21.52 (0.93)	19.73 (0.94)	
No	77.57 (0.56)	73.02 (1.00)	76.35 (1.01)	78.48 (0.93)	80.27 (0.94)	
Lipid-lowing agent, % (SE)						0.3858
Yes	16.57 (0.47)	15.38 (0.91)	16.88 (0.82)	16.21 (0.88)	17.32 (0.77)	
No	83.43 (0.47)	84.62 (0.91)	83.12 (0.82)	83.79 (0.88)	82.68 (0.77)	
CKM Stages, % (SE)						< 0.0001
0	13.76 (0.45)	7.65 (0.60)	11.41 (0.78)	14.05 (0.73)	18.67 (0.87)	
1	30.54 (0.56)	26.46 (1.04)	30.88 (1.03)	31.58 (1.07)	31.75 (0.99)	
2	42.77 (0.60)	47.98 (1.26)	43.71 (1.04)	42.56 (1.14)	39.33 (0.96)	
3	4.86 (0.20)	7.03 (0.48)	5.51 (0.45)	4.34 (0.35)	3.59 (0.30)	
4	8.07 (0.31)	10.89 (0.74)	8.50 (0.60)	7.47 (0.52)	6.66 (0.44)	
Outcomes, % (SE)						
All-cause mortality	7.63 (0.34)	11.81 (0.68)	8.36 (0.57)	6.43 (0.45)	5.74 (0.43)	< 0.0001
Cardiovascular mortality	2.16 (0.14)	3.35 (0.35)	2.75 (0.32)	1.79 (0.20)	1.38 (0.15)	< 0.0001

Values are presented as the mean ± standard error (SE) unless stated otherwise. BMI, body mass index; PIR, poverty-to-income rations; TG, triglyceride; TC, total cholesterol; HDL-C, high density lipoprotein cholesterol; LDL-C, low density lipoprotein cholesterol; FBG, fasting blood glucose; HbA1c, glycohemoglobin; eGFR, estimated glomerular filtration rate; ALT, alanine aminotransferase; AST, aspartate aminotransferase.

### Association between AGR and mortality

Table 2 presents the association between AGR and all-causes and CVD mortality. After full adjustment, the hazard rations (HR) for all-cause mortality across increasing AGR quartiles were: 1.00 (reference), 0.67 (95% CI: 0.56–0.79), 0.54 (95% CI: 0.44–0.66), and 0.55 (95% CI: 0.45–0.67), respectively, for all-cause mortality (P for trend < 0.0001). For CVD mortality, the corresponding HR were: 1.00 (reference), 0.79 (95% CI: 0.58–1.09), 0.49 (95% CI: 0.34–0.71), and 0.47 (95% CI: 0.34–0.64), respectively, for CVD mortality (P for trend < 0.0001).

**Table 2:** HR (95%CI) for all-cause and CVD mortality according to AGR among participants with CKM syndrome in NHANES 2003–2018.

Outcomes	AGR					
	Q1 (0.40–1.26)	Q2 (1.27–1.44)	Q3 (1.45–1.63)	Q4 (1.64–5.88)	P trend	AGR continuous
All-cause mortality						
Number of deaths (%)	593 (14.98)	444 (11.27)	377 (9.37)	331 (8.33)		1745 (10.98)
Model 1	1.00	0.67 (0.57, 0.78)	0.50 (0.42, 0.61)	0.47 (0.40, 0.55)	< 0.0001	0.31 (0.24, 0.40)
P-value		< 0.0001	< 0.0001	< 0.0001		< 0.0001
Model 2	1.00	0.65 (0.55, 0.78)	0.49 (0.40, 0.60)	0.49 (0.40, 0.59)	< 0.0001	0.32 (0.24, 0.43)
P-value		< 0.0001	< 0.0001	< 0.0001		< 0.0001
Model 3	1.00	0.67 (0.56, 0.79)	0.54 (0.44, 0.66)	0.55 (0.45, 0.67)	< 0.0001	0.38 (0.28, 0.49)
P-value		< 0.0001	< 0.0001	< 0.0001		< 0.0001
CVD mortality						
Number of deaths (%)	175 (4.42)	149 (3.78)	118 (2.93)	92 (2.32)		534 (3.36)
Model 1	1.00	0.76 (0.57, 1.01)	0.48 (0.35, 0.65)	0.38 (0.30, 0.50)	< 0.0001	0.23 (0.15, 0.35)
P-value		0.0595	< 0.0001	< 0.0001		< 0.0001
Model 2	1.00	0.77 (0.56, 1.06)	0.45 (0.32, 0.64)	0.41 (0.30, 0.56)	< 0.0001	0.22 (0.13, 0.39)
P-value		0.1046	< 0.0001	< 0.0001		< 0.0001
Model 3	1.00	0.79 (0.58, 1.09)	0.49 (0.34, 0.71)	0.47 (0.34, 0.64)	< 0.0001	0.27 (0.16, 0.44)
P-value		0.1606	0.0002	< 0.0001		< 0.0001

Model 1: Non-adjusted; Model 2: Adjusted for age, sex and race; Model 3: Adjusted for age, sex, race, education level, marital status, diabetes mellitus, hypertension, FBG, and HbA1c.

### Nonlinear association detection

**Figure 2:**
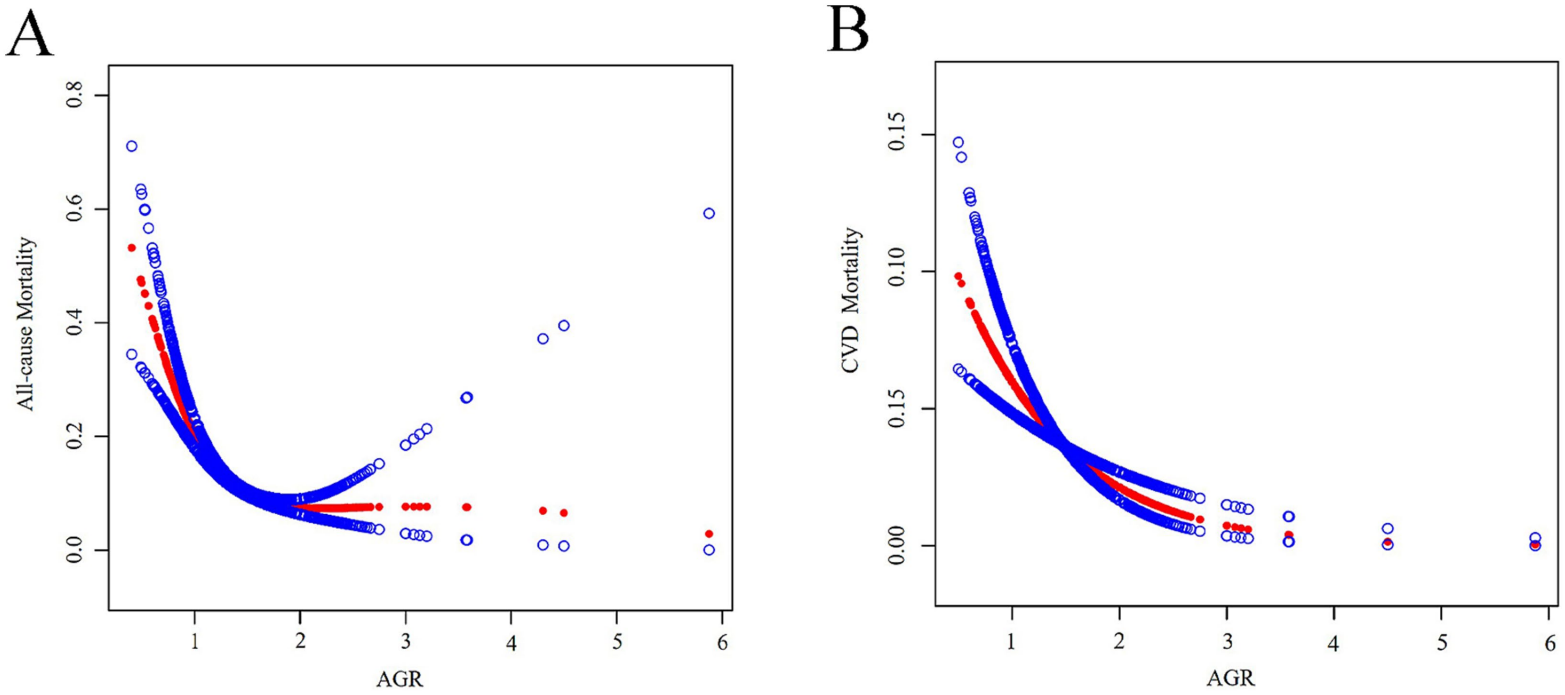
**(A)** A generalized additive model that establishes the nonlinear relationship between AGR and all-cause mortality. **(B)** A generalized additive model that establishes the linear relationship between AGR and CVD mortality. Adjusted for age, sex, race, education level, marital status, diabetes mellitus, hypertension, FBG, and HbA1c.

Figure 2 illustrates the results from smooth curve fitting analysis. A nonlinear relationship was observed between AGR and all-cause mortality (Figure 2A), while a linear association was identified for AGR and CVD mortality (Figure 2B). To further examine this nonlinearity, we applied a two-piecewise Cox hazards regression model. As shown in Table 3, the inflection point for AGR in relation to all-cause mortality was 1.26. Below this threshold (AGR < 1.26), each 1-unit increase in AGR was associated with a 93% reduction in the risk of all-cause mortality (HR = 0.07, 95% CI: 0.05–0.12).

**Table 3:** Threshold effect analysis of AGR on all-cause mortality among participants with CKM syndrome.

Outcomes	Adjusted HR (95% CI), *P*-value
All-cause mortality	
Fitting by the standard linear model	0.41 (0.33, 0.50) < 0.0001
Fitting by the two-piecewise linear model	
Inflection point	1.26
AGR < 1.26	0.07 (0.05, 0.12) < 0.0001
zAGR ≥ 1.26	0.79 (0.62, 1.01) 0.0653
P for log-likelihood ratio	< 0.001

Adjusted for age, sex, race, education level, marital status, diabetes mellitus, hypertension, FBG, and HbA1c.

### Subgroup analysis

Tables 4, 5 present the results of subgroup analyses for the association between AGR and both all-cause and CVD mortality. Subgroups were categorized by age (< 60 years old or ≥ 60 years old), sex, race, education level, BMI (< 25, 25–29.9, and ≥ 30 kg/m^2^), history of diabetes mellitus or hypertension, smoking status, alcohol intake, PIR (< 1.3, 1.3–3.0, and ≥ 3.0), and CKM stage. The inverse association between AGR and all-cause mortality was stronger among subgroups defined by age, race, smoking status, and CKM stage (all p for interaction < 0.05). For CVD mortality, stronger associations were observed by age, education level, and smoking status (all p for interaction < 0.05). Association in other subgroups remained consistent (all p for interaction > 0.05).

**Table 4:** Subgroup analyses for the relationship between AGR and all-cause mortality.

Subgroups	HR (95%CI)	*P*	P for interaction
Age (years)			< 0.0001
< 60	0.14 (0.09, 0.22)	< 0.0001	
≥ 60	0.45 (0.36, 0.56)	< 0.0001	
Sex			0.3234
Male	0.38 (0.29, 0.48)	< 0.0001	
Female	0.46 (0.34, 0.61)	< 0.0001	
Race			0.0040
Mexican American	0.17 (0.09, 0.35)	< 0.0001	
Non-Hispanic White	0.51 (0.40, 0.64)	< 0.0001	
Non-Hispanic Black	0.27 (0.16, 0.46)	< 0.0001	
Other Race	0.27 (0.14, 0.51)	< 0.0001	
Education level			0.2875
Less than 9th grade	0.39 (0.25, 0.62)	< 0.0001	
9-11th grade	0.39 (0.25, 0.62)	< 0.0001	
High school graduate	0.47 (0.33, 0.67)	< 0.0001	
College degree	0.32 (0.22, 0.48)	< 0.0001	
College and above	0.49 (0.31, 0.79)	0.0033	
BMI (kg/m^2^)			0.2533
< 25	0.42 (0.30, 0.57)	< 0.0001	
25–30	0.52 (0.38, 0.71)	< 0.0001	
≥ 30	0.27 (0.19, 0.39)	< 0.0001	
Diabetes mellitus			0.2524
Yes	0.34 (0.23, 0.49)	< 0.0001	
No	0.43 (0.35, 0.54)	< 0.0001	
Hypertension			0.2784
Yes	0.44 (0.35, 0.57)	< 0.0001	
No	0.36 (0.27, 0.49)	< 0.0001	
Smoking status			0.0005
Every day	0.21 (0.14, 0.31)	< 0.0001	
Some day	0.46 (0.34, 0.62)	< 0.0001	
Not at all	0.53 (0.39, 0.71)	< 0.0001	
Alcohol user			0.3231
Yes	0.26 (0.17, 0.40)	< 0.0001	
No	0.45 (0.36, 0.56)	< 0.0001	
PIR			0.5806
< 1.3	0.39 (0.27, 0.55)	< 0.0001	
1.3–3.0	0.42 (0.31, 0.58)	< 0.0001	
≥ 3.0	0.50 (0.35, 0.71)	0.0001	
CKM stage			0.0399
Stage 0	0.27 (0.09, 0.86)	0.0266	
Stage 1	0.24 (0.14, 0.41)	< 0.0001	
Stage 2	0.40 (0.26, 0.61)	< 0.0001	
Stage 3	0.61 (0.44, 0.85)	0.0030	
Stage 4	0.43 (0.31, 0.59)	< 0.0001	

Adjusted for age, sex, race, education level, marital status, diabetes mellitus, hypertension, FBG, and HbA1c.

**Table 5:** Subgroup analyses for the relationship between AGR and CVD mortality.

Subgroups	HR (95%CI)	*P*	P for interaction
Age (years)			0.0002
< 60	0.07 (0.03, 0.17)	< 0.0001	
≥ 60	0.41 (0.28, 0.60)	< 0.0001	
Sex			0.4617
Male	0.36 (0.23, 0.56)	< 0.0001	
Female	0.46 (0.27, 0.81)	0.0073	
Race			0.2138
Mexican American	0.22 (0.06, 0.78)	0.0194	
Non-Hispanic White	0.51 (0.33, 0.79)	0.0027	
Non-Hispanic Black	0.20 (0.08, 0.49)	0.0005	
Other Race	0.34 (0.11, 1.07)	0.0659	
Education level			0.0073
Less than 9th grade	0.45 (0.39, 0.53)	< 0.0001	
9-11th grade	0.63 (0.54, 0.74)	< 0.0001	
High school graduate	0.43 (0.37, 0.49)	< 0.0001	
College degree	0.23 (0.20, 0.27)	< 0.0001	
College and above	0.44 (0.37, 0.51)	< 0.0001	
BMI (kg/m^2^)			0.0994
< 25	0.53 (0.29, 0.96)	0.0333	
25–30	0.48 (0.27, 0.86)	0.00134	
≥ 30	0.23 (0.12, 0.42)	< 0.0001	
Diabetes mellitus			0.4266
Yes	0.31 (0.16, 0.62)	0.0008	
No	0.43 (0.28, 0.65)	< 0.0001	
Hypertension			0.9558
Yes	0.40 (0.25, 0.62)	< 0.0001	
No	0.39 (0.22, 0.70)	0.0015	
Smoking status			0.0075
Every day	0.12 (0.05, 0.28)	< 0.0001	
Some day	0.48 (0.27, 0.85)	0.0125	
Not at all	0.53 (0.32, 0.87)	0.0129	
Alcohol user			0.5437
Yes	0.30 (0.12, 0.78)	0.0139	
No	0.41 (0.28, 0.60)	< 0.0001	
PIR			0.9390
< 1.3	0.37 (0.19, 0.73)	0.0039	
1.3–3.0	0.43 (0.24, 0.77)	0.0048	
≥ 3.0	0.43 (0.23, 0.80)	0.0085	
CKM stage			0.4044
Stage 0	1.34 (0.06, 1.78)	0.8565	
Stage 1	0.17 (0.05, 0.54)	0.0027	
Stage 2	0.33 (0.14, 0.79)	0.0128	
Stage 3	0.51 (0.28,0.93)	0.0285	
Stage 4	0.48 (0.28, 0.83)	0.0083	

Adjusted for age, sex, race, education level, marital status, diabetes mellitus, hypertension, FBG, and HbA1c.

### Sensitivity analysis

Robustness of the primary findings was confirmed through multiple sensitivity analyses. First, exclusion of participants who died the first 2 years of follow-up did not materially change the associations (Supplementary Table S10). Second, excluding participants with baseline CVD or cancer yielded similar results (Supplementary Table S11). Third, removing those using hypoglycemic, antihypertensive, or lipid-lowering medications at baseline also had no significant effect on the observed relationships (Supplementary Table S12). Finally, the analysis was restricted to participants in CKM stages 3–4, which yielded consistent findings (Supplementary Table S13).

## Discussion

This study represents the first comprehensive investigation of the association between AGR and the risks of all-cause and CVD mortality in a large cohort of participants with CKM syndrome. Our findings provide compelling evidence that higher AGR levels are independently associated with substantially lower risks of both all-cause and CVD mortality. Critically, we identified a nonlinear relationship between AGR and all-cause mortality, whereas the association with CVD mortality followed a linear trajectory. Subgroup analyses revealed that the protective association of AGR was particularly pronounced among younger individuals (< 60 years), daily smokers, Mexican Americans, college-educated participants, and those in stage 1 of CKM. These associations remained robust across a series of extensive stratified and sensitivity analyses.

While albumin and globulin levels individually reflect inflammation and nutritional status, their clinical interpretation is often confounded by acute-phase reactions, fluid shifts, and organ dysfunction. By integrating these two measures, the AGR provides a more stable and physiologically meaningful biomarker. Prior studies have established AGR as a diagnostic and prognostic tool in infectious diseases and a broad array of malignancies, with lower AGR consistently predicting poorer survival in colorectal, gastric, breast, head and neck cancers (24, 28–30). For instance, Li et al. demonstrated that preoperative AGR effectively predicted long-term survival in colorectal cancer patients (24). Beyond oncology, evidence remains scarce but provocative. Otaki et al. reported that AGR independently predicted cardiac events and rehospitalizations among women with heart failure with preserved ejection fraction (31). Similarly, Azab et al. identified AGR < 1.34 as a potent independent predictor of long-term mortality among non-ST elevation myocardial infarction patients, outperforming albumin alone (32). Studies in acute ischemic stroke and diabetic populations further corroborate AGR’s prognostic potential (23, 33). Yet, remarkably, no prior research had examined its role in the high-risk CKM syndrome population. Our analysis of NHANES 2003–2018 data addresses this critical knowledge gap. We demonstrate for the first time that AGR exhibits a nonlinear association with all-cause mortality and a linear association with CVD mortality in individuals with CKM syndrome, even after rigorous adjustment for potential confounders. Mechanistically, CKM syndrome is characterized by systemic inflammation and progressive malnutrition, both of which disrupt protein homeostasis and lead to reduced AGR. Our findings suggest that this inflammatory—nutritional imbalance critically underpins the observed increase in mortality risk.

Stratified analyses uncovered notable effect modifications. The inverse association between AGR and all-cause mortality was most evident among younger adults, daily smokers, Mexican Americans, and individuals in CKM stage 1; while its relationship with CVD mortality was strongest among college graduates, younger individuals, and daily smokers. These patterns align with existing literature: Zeng et al. reported enhanced AGR predictive power in younger CKD patients (34), and He et al. confirmed AGR’s utility in diabetic adults under 60 (23). Furthermore, recent research indicates that both daily and non-daily smokers face elevated all-cause and CVD mortality compared to never-smokers (35, 36). Mexican Americans exhibit disproportionate burdens of obesity, metabolic syndrome, and under-treatment hypertension—factors that likely amplify the prognostic significance of AGR (37). Although higher education typically confers health benefits, emerging evidence suggests that lifestyle factors such as sedentary behavior and chronic stress among highly educated individuals may paradoxically increase CVD risk through heightened sympathetic activity and endothelial dysfunction (38, 39). While clinical manifestations in CKM stage 1 are relatively mild, chronic low-grade inflammation is already activated. A decrease in AGR may serve as a sensitive marker of this “subclinical inflammation.” Moreover, during early CKM stage 1, traditional biomarkers such as eGFR and proteinuria may remain within normal ranges. As AGR integrates signals from the nutrition-inflammation-immunity axis, it may offer superior predictive capability in this early stage of disease.

By leveraging the large, nationally representative NHANES 2003–2018 cohort, our study transcends previous disease-specific investigations and firmly establishes AGR as a robust, generalizable prognostic marker across a diverse U. S. adult population. Importantly, the discovery of a nonlinear association between AGR and all-cause mortality introduces a crucial new dimension to AGR’s clinical relevance: once AGR falls below a critical threshold, mortality risk escalates sharply. This nuanced understanding moves beyond the simplistic linear associations previously described (23, 40), offering a more sophisticated model of AGR’s impact on survival outcomes. Clinically, these findings suggest that integrating AGR into routine risk stratification models for CKM syndrome could substantially enhance early identification of high-risk individuals and guide targeted preventive interventions. Future prospective studies are warranted to validate these observations and to elucidate the biological mechanisms linking AGR dynamics with CKM progression and outcomes. Ultimately, the incorporation of AGR into precision medicine frameworks holds transformative potential for improving prognostication and tailoring interventions in this vulnerable population.

Several mechanisms may underlie the observed association between lower AGR and poorer prognosis. We hypothesize that this relationship is primarily driven by pro-inflammatory and pro-oxidative pathways. Serum albumin plays diverse physiological roles, including anticoagulant, antioxidant and anti-inflammatory functions, as well as preserving vascular integrity (41–43). It also binds and transports inflammatory mediators, modulating both systemic and tissue-specific inflammation while mitigating oxidative stress.

Chronic inflammation and oxidative stress are central to the pathogenesis of CKM syndrome (7, 16). Inflammatory cytokines released by activated macrophages redirect hepatic protein synthesis from albumin toward acute-phase proteins, reducing serum albumin levels (44). Low serum albumin has emerged as a strong, independent predictor of mortality. For example, Li et al. reported that each 1 g/dL decrease in serum albumin was associated with a 3.85-fold increase in CVD mortality (45). Similarly, Hartopo et al. found that hypoalbuminemia predicted a 2.8-fold higher risk of adverse outcomes in acute coronary syndrome (46).

A prothrombotic state may represent an additional mechanism linking low AGR to mortality. Albumin inhibits thromboxane A₂ synthesis by modulating free arachidonic acid levels (47). Tanahashi et al. showed that lower AGR levels were linked to enhanced erythrocyte aggregability in patients with cerebrovascular disease (48). In patients with chronic renal failure undergoing peritoneal dialysis, correction of hypoalbuminemia through albumin administration was shown to reduce platelet aggregation (49).

Our findings position AGR as a valuable adjunct to traditional risk scores such as atherosclerotic cardiovascular disease (ASCVD) and CKD staging. While conventional models like the ASCVD score focus on established risk factors—age, cholesterol, blood pressure, and smoking—they fail to capture systemic inflammation and nutritional status, both of which are now recognized as critical contributors to cardiovascular and all-cause mortality. Similarly, CKD staging relies on eGFR and albuminuria, but does not account for the immunoinflammatory milieu that underlies disease progression and adverse outcomes.

As a composite of hypoalbuminemia and hyperglobulinemia, AGR may reveal high-risk individuals missed by traditional models. In our cohort, patients classified as stage 0 with low AGR still faced elevated mortality risk—likely driven by subclinical inflammation or malnutrition. Conversely, AGR can further stratify risk among individuals already classified as high-risk. Prior research shows that AGR < 1.34 is associated with significantly higher long-term mortality following non-ST-segment elevation myocardial infarction (32). Additionally, AGR has been identified as an independent predictor of long-term mortality in CKD patients, especially in males under age 65 (34). Incorporating AGR into existing risk models may improve predictive accuracy, particularly in populations with chronic conditions or age-related vulnerability. Prospective studies are needed to validate the incremental prognostic value of AGR within established clinical scoring systems.

This study possesses several notable strengths. First, our analysis leverages NHANES data, a large, nationally representative cohort with rigorous methodological standardization, enhancing the generalizability of our findings. Second, our confounder adjustments were guided by prior research on AGR and mortality, as well as multiple sensitivity analyses were conducted, bolstering the robustness of our results. Finally, we are the first to employ a smooth curve fitting model to assess the relationship between AGR and mortality, introducing a novel analytical perspective to this field.

However, several limitations must be acknowledged. The cross-sectional study design precludes causal inference, emphasizing the need for longitudinal studies to validate our findings. Additionally, AGR was measured only at baseline, which may not fully capture their temporal fluctuations and evolving associations with mortality risk. Despite rigorous confounder adjustments, residual confounding from unmeasured variables cannot be entirely excluded. Lastly, as our data are derived from a U. S. cohort, extrapolating our findings to other populations requires further epidemiological validation. Future studies should aim to refine risk stratification models incorporating AGR, potentially informing personalized strategies for CKM management.

## Conclusion

Our study confirms that higher AGR is independently and robustly associated with lower risks of both all-cause and CVD mortality, underscoring the pivotal roles of inflammation and nutritional status in the progression of CKM syndrome. As a readily available clinical measure, AGR offers a practical biomarker for enhanced risk stratification and early patient identification. Future research should prioritize integrating AGR into multivariable prognostic models, validating its incremental value for guiding targeted therapeutic strategies and ultimately improving patient outcomes.

## Data Availability

The datasets presented in this study can be found in online repositories. The names of the repository/repositories and accession number(s) can be found at: The data of this article is accessible at NHANES website: https://www.cdc.gov/nchs/nhanes/?CDC.

## Footnotes

^1^ https://www.cdc.gov/nchs/nhanes/

^2^ www.empowerstats.com

^3^ http://www.R-project.org

## References

[1] Roth GA Mensah GA Johnson CO Addolorato G Ammirati E Baddour LM. Global burden of cardiovascular diseases and risk factors, 1990-2019: update from the GBD 2019 study. J Am Coll Cardiol. (2020) 76:2982–3021. doi: 10.1016/j.jacc.2020.11.010, PMID: 33309175 PMC7755038

[2] Global, 2021 Diabetes Collaborators. Global, regional, and national burden of diabetes from 1990 to 2021, with projections of prevalence to 2050: a systematic analysis for the global burden of disease study 2021. Lance. (2023) 402:203–34. doi: 10.1016/S0140-6736(23)01301-6 PMC10364581 37356446

[3] Deng Y Li N Wu Y Wang M Yang S Zheng Y. Global, regional, and National Burden of diabetes-related chronic kidney disease from 1990 to 2019. Front Endocrinol. (2021) 12:672350. doi: 10.3389/fendo.2021.672350, PMID: 34276558 PMC8281340

[4] Aggarwal R Ostrominski JW Vaduganathan M. Prevalence of cardiovascular-kidney-metabolic syndrome stages in US adults, 2011-2020. JAMA. (2024) 331:1858–60. doi: 10.1001/jama.2024.6892, PMID: 38717747 PMC11079779

[5] Minhas AMK Mathew RO Sperling LS Nambi V Virani SS Navaneethan SD. Prevalence of the cardiovascular-kidney-metabolic syndrome in the United States. J Am Coll Cardiol. (2024) 83:1824–6. doi: 10.1016/j.jacc.2024.03.368 38583160

[6] Kim JE Joo J Kuku KO Downie C Hashemian M Powell-Wiley TM. Prevalence, disparities, and mortality of cardiovascular-kidney-metabolic syndrome in US adults, 2011-2018. Am J Med. (2025) S0002-9343:00063–4. doi: 10.1016/j.amjmed.2025.01.031 PMC12092198 39909293

[7] Ndumele CE Neeland IJ Tuttle KR Chow SL Mathew RO Khan SS. A synopsis of the evidence for the science and clinical Management of Cardiovascular-Kidney-Metabolic (CKM) syndrome: a scientific statement from the American Heart Association. Circulation. (2023) 148:1636–64. doi: 10.1161/CIR.0000000000001186, PMID: 37807920

[8] Claudel SE Schmidt IM Waikar SS Verma A. Cumulative incidence of mortality associated with cardiovascular-kidney-metabolic (CKM) syndrome. J Am Soc Nephrol. (2025):11. doi: 10.1681/ASN.0000000637 PMC12187233 39932805

[9] Ostrominski JW Arnold SV Butler J Fonarow GC Hirsch JS Palli SR. Prevalence and overlap of cardiac, renal, and metabolic conditions in US adults, 1999-2020. JAMA Cardiol. (2023) 8:1050–60. doi: 10.1001/jamacardio.2023.3241, PMID: 37755728 PMC10535010

[10] Center For Cardiovascular Diseases The Writing Committee Of The Report On Cardiovascular Health And Diseases In China N. Report on cardiovascular health and diseases in China. Biomed Environ Sci. (2023) 2024:949–92. doi: 10.3967/bes2024.162 39401992

[11] Yamaguchi M Ito M Sugiyama H Iwagaitsu S Nobata H Kinashi H. Malnutrition according to the GLIM criteria with kidney dysfunction is associated with increased mortality in hospitalized patients with cardiovascular disease: a retrospective cohort study. Clini Nutr ESPEN. (2023) 55:167–73. doi: 10.1016/j.clnesp.2023.02.029, PMID: 37202041

[12] Sabbouh T Torbey MT. Malnutrition in stroke patients: risk factors, assessment, and management. Neurocrit Care. (2017) 29:374–84. doi: 10.1007/s12028-017-0436-1 PMC5809242 28799021

[13] Lu Y Nyunt MSZ Gao Q Gwee X Chua DQ Yap KB. Malnutrition risk and kidney function and decline in community-dwelling older adults. J Ren Nutr. (2022) 32:560–8. doi: 10.1053/j.jrn.2021.09.002, PMID: 35300925

[14] Liu P Tian H Ji T Zhong T Gao L Chen L. Predictive value of malnutrition, identified via different nutritional screening or assessment tools, for functional outcomes in patients with stroke: a systematic review and Meta-analysis. Nutrients. (2023) 15:3280. doi: 10.3390/nu15143280, PMID: 37513698 PMC10383200

[15] Liang L Zhao X Huang L Tian P Huang B Feng J. Prevalence and prognostic importance of malnutrition, as assessed by four different scoring systems, in elder patients with heart failure. Nutr Metab Cardiovasc Dis. (2023) 33:978–86. doi: 10.1016/j.numecd.2023.01.004, PMID: 36710105

[16] Sebastian SA Padda I Johal G. Cardiovascular-kidney-metabolic (CKM) syndrome: a state-of-the-art review. Curr Probl Cardiol. (2024) 49:102344. doi: 10.1016/j.cpcardiol.2023.102344, PMID: 38103820

[17] Cao Y Wang W Xie S Xu Y Lin Z. Joint association of the inflammatory marker and cardiovascular-kidney-metabolic syndrome stages with all-cause and cardiovascular disease mortality: a national prospective study. BMC Public Health. (2025) 25:10. doi: 10.1186/s12889-024-21131-2 39748335 PMC11697861

[18] Li W Shen C Kong W Zhou X Fan H Zhang Y. Association between the triglyceride glucose-body mass index and future cardiovascular disease risk in a population with cardiovascular-kidney-metabolic syndrome stage 0-3: a nationwide prospective cohort study. Cardiovasc Diabetol. (2024) 23:292. doi: 10.1186/s12933-024-02352-6 39113004 PMC11308445

[19] Song M Graubard BI Rabkin CS Engels EA. Neutrophil-to-lymphocyte ratio and mortality in the United States general population. Sci Rep. (2021) 11:464. doi: 10.1038/s41598-020-79431-7 33431958 PMC7801737

[20] Wang A Zhang Y Xia G Tian X Zuo Y Chen P. Association of serum albumin to globulin ratio with outcomes in acute ischemic stroke. CNS Neurosci Ther. (2023) 29:1357–67. doi: 10.1111/cns.14108, PMID: 36794538 PMC10068453

[21] Wang SF Wu TT Zheng YY Hou XG Yang HT Yang Y. Serum globulin to albumin ratio as a novel predictor of adverse clinical outcomes in coronary artery disease patients who underwent PCI. Rev Cardiovasc Med. (2023) 24:278. doi: 10.31083/j.rcm2410278, PMID: 39077558 PMC11273180

[22] Peng F Sun L Chen T Zhu Y Zhou W Li P. Albumin-globulin ratio and mortality in patients on peritoneal dialysis: a retrospective study. BMC Nephrol. (2020) 21:51. doi: 10.1186/s12882-020-1707-1 32059708 PMC7023751

[23] Wen H Niu X Yu R Zhao R Wang Q Sun N. Association of Serum AGR with all-cause and cause-specific mortality among individuals with diabetes. J Clin Endocrinol Metab. (2025) 110:e266–75. doi: 10.1210/clinem/dgae215, PMID: 38571296

[24] Li J Zhu N Wang C You L Guo W Yuan Z. Preoperative albumin-to-globulin ratio and prognostic nutritional index predict the prognosis of colorectal cancer: a retrospective study. Sci Rep. (2023) 13:17272. doi: 10.1038/s41598-023-43391-5 37828259 PMC10570287

[25] Levey AS Stevens LA Schmid CH Zhang YL Castro AF Feldman HI. A new equation to estimate glomerular filtration rate. Ann Intern Med. (2009) 150:604–12. doi: 10.7326/0003-4819-150-9-200905050-00006, PMID: 19414839 PMC2763564

[26] Khan SS Coresh J Pencina MJ Ndumele CE Rangaswami J Chow SL. Novel prediction equations for absolute risk assessment of Total cardiovascular disease incorporating cardiovascular-kidney-metabolic health: a scientific statement from the American Heart Association. Circulation. (2023) 148:1982–2004. doi: 10.1161/CIR.0000000000001191, PMID: 37947094

[27] KDIGO. Clinical practice guideline for diabetes Management in Chronic Kidney Disease. Kidney Int. (2020) 2020:S1–S115. doi: 10.1016/j.kint.2020.06.019 32998798

[28] Xuan Q Yang Y Ji H Tang S Zhao J Shao J. Combination of the preoperative albumin to globulin ratio and neutrophil to lymphocyte ratio as a novel prognostic factor in patients with triple negative breast cancer. Cancer Manag Res. (2019) 11:5125–31. doi: 10.2147/CMAR.S195324 31213922 PMC6549418

[29] Wei C Yu Z Wang G Zhou Y Tian L. Low pretreatment albumin-to-globulin ratio predicts poor prognosis in gastric Cancer: insight from a Meta-analysis. Front Oncol. (2020) 10:623046. doi: 10.3389/fonc.2020.623046, PMID: 33575220 PMC7870866

[30] Wang YT Kuo LT Lai CH Tsai YH Lee YC Hsu CM. Low pretreatment albumin-to-globulin ratios predict poor survival outcomes in patients with head and neck Cancer: a systematic review and Meta-analysis. J Cancer. (2023) 14:281–9. doi: 10.7150/jca.80955, PMID: 36741261 PMC9891875

[31] Otaki Y Shimizu M Watanabe T Tachibana S Sato J Kobayashi Y. Albumin-to-globulin ratio predicts clinical outcomes of heart failure with preserved ejection fraction in women. Heart Vessel. (2022) 37:1829–40. doi: 10.1007/s00380-022-02087-y, PMID: 35596031

[32] Azab B Bibawy J Harris K Khoueiry G Akerman M Selim J. Value of albumin-globulin ratio as a predictor of all-cause mortality after non-ST elevation myocardial infarction. Angiology. (2013) 64:137–45. doi: 10.1177/0003319712436577, PMID: 22345150

[33] Tan X Lv C Lu C Luo Y Mei ZG. Association between serum a/G ratio and stroke: data from NHANES 2009-2020. Front Nutr. (2025) 12:1512165. doi: 10.3389/fnut.2025.1512165 40070476 PMC11895003

[34] Zeng M Liu Y Liu F Peng Y Sun L Xiao L. Association between albumin-to-globulin ratio and long-term mortality in patients with chronic kidney disease: a cohort study. Int Urol Nephrol. (2020) 52:1103–15. doi: 10.1007/s11255-020-02453-7, PMID: 32405697

[35] Christensen CH Rostron B Cosgrove C Altekruse SF Hartman AM Gibson JT. Association of Cigarette, cigar, and pipe use with mortality risk in the US population. JAMA Intern Med. (2018) 178:469–76. doi: 10.1001/jamainternmed.2017.8625, PMID: 29459935 PMC5876825

[36] Inoue-Choi M Shiels MS McNeel TS Graubard BI Hatsukami D Freedman ND. Contemporary associations of exclusive cigarette, cigar, pipe, and smokeless tobacco use with overall and cause-specific mortality in the United States. JNCI Cancer Spectr. (2019) 3:pkz 036. doi: 10.1093/jncics/pkz036 PMC6620791 31321380

[37] Tsao CW Aday AW Almarzooq ZI Anderson CAM Arora P Avery CL. Heart disease and stroke Statistics-2023 update: a report from the American Heart Association. Circulation. (2023) 147:e93–e621. doi: 10.1161/CIR.0000000000001123, PMID: 36695182 PMC12135016

[38] Lavie CJ Ozemek C Carbone S Katzmarzyk PT Blair SN. Sedentary behavior, exercise, and cardiovascular health. Circ Res. (2019) 124:799–815. doi: 10.1161/CIRCRESAHA.118.312669, PMID: 30817262

[39] Vaccarino V Bremner JD. Stress and cardiovascular disease: an update. Nat Rev Cardiol. (2024) 21:603–16. doi: 10.1038/s41569-024-01024-y 38698183 PMC11872152

[40] Xu Q Wang J Li H Chen X. A study investigating how the albumin-globulin ratio relates to depression risk within U.S. adults: a cross-sectional analysis. Front Nutr. (2024) 11:1453044. doi: 10.3389/fnut.2024.1453044 39421614 PMC11484099

[41] Roche M Rondeau P Singh NR Tarnus E Bourdon E. The antioxidant properties of serum albumin. FEBS Lett. (2008) 582:1783–7. doi: 10.1016/j.febslet.2008.04.057, PMID: 18474236

[42] Huang CY Liou SY Kuo WW Wu HC Chang YL Chen TS. Chemiluminescence analysis of antioxidant capacity for serum albumin isolated from healthy or uremic volunteers. Luminescence. (2016) 31:1474–8. doi: 10.1002/bio.3132, PMID: 27062681

[43] Anraku M Chuang VT Maruyama T Otagiri M. Redox properties of serum albumin. Biochim Biophys Acta. (2013) 1830:5465–72. doi: 10.1016/j.bbagen.2013.04.036 23644037

[44] Niedziela JT Hudzik B Szygula-Jurkiewicz B Nowak JU Polonski L Gasior M. Albumin-to-globulin ratio as an independent predictor of mortality in chronic heart failure. Biomark Med. (2018) 12:749–57. doi: 10.2217/bmm-2017-0378, PMID: 29865856

[45] Li X Zhang Y He Y Li KX Xu RN Wang H. J-shaped association between serum albumin levels and long-term mortality of cardiovascular disease: experience in National Health and nutrition examination survey (2011-2014). Front Cardiovasc Med. (2022) 9:1073120. doi: 10.3389/fcvm.2022.1073120 36523355 PMC9745145

[46] Hartopo AB Gharini PP Setianto BY. Low serum albumin levels and in-hospital adverse outcomes in acute coronary syndrome. Int Heart J. (2010) 51:221–6. doi: 10.1536/ihj.51.221, PMID: 20716836

[47] Gresele P Deckmyn H Huybrechts E Vermylen J. Serum albumin enhances the impairment of platelet aggregation with thromboxane synthase inhibition by increasing the formation of prostaglandin D2. Biochem Pharmacol. (1984) 33:2083–8. doi: 10.1016/0006-2952(84)90577-X, PMID: 6430299

[48] Tanahashi N Gotoh F Tomita M Shinohara T Terayama Y Mihara B. Enhanced erythrocyte aggregability in occlusive cerebrovascular disease. Stroke. (1989) 20:1202–7. doi: 10.1161/01.STR.20.9.1202, PMID: 2475922

[49] Sloand EM Bern MM Kaldany A. Effect on platelet function of hypoalbuminemia in peritoneal dialysis. Thromb Res. (1986) 44:419–25. doi: 10.1016/0049-3848(86)90320-8, PMID: 3798407

