# Supplementary material for: Association between albumin to globulin ratio and all-cause and cardiovascular mortality among individuals with cardiovascular-kidney-metabolic syndrome: results from NHANES 2003 to 2018
Source: Front Nutr. 2025 Jul 3;12:1622590. doi: 10.3389/fnut.2025.1622590 (PMC12267012; doi:10.3389/fnut.2025.1622590)
Supplement: Supplementary file 1 [file Table_1.DOCX]

**Supplementary material**

**Table S1** Definitions of CKM conditions

| CKM conditions | CKM indicators | Threshold for CKM indicators |
| --- | --- | --- |
| CVD | clinical CVD | History of CHF, CHD, AP, heart attack, or stroke |
|  | subclinical CVD | Any of the following criterion is met:  a) Very high -risk CKD in KDIGO classification: UACR ≥ 300mg/g and eGFR ≤ 45-59 ml/min/1.73m^2^, UACR ≥ 30mg/g and eGFR ≤ 30-44 ml/min/1.73m^2^, or eGFR ≤ 29 ml/min/1.73m^2^  b) Predicted 10-year CVD risk ≥ 20% |
| CKD | CKD | Moderate-to-high -risk CKD in KDIGO classification: UACR ≥ 30mg/g and eGFR ≥ 60ml/min/1.73m^2^, UACR < 300mg/g and eGFR ≤ 45-59 ml/min/1.73m^2^, or UACR < 30mg/g and eGFR ≤ 30-44 ml/min/1.73m^2^ |
| Metabolic disorders | Overweight | BMI ≥ 25kg/m^2^ (or ≥ 23kg/m^2^ if Asian ancestry) |
|  | Abdominal obesity | WC ≥ 88/102 cm if female/male (or if Asian ancestry ≥ 80/90 cm in female/male) |
|  | Prediabetes | FBG ≥ 100-124mg/dL or HbA1c ≥ 5.7%-6.4% and without self-reported diagnosis of diabetes, use of insulin, or oral hypoglycemic agents |
|  | Diabetes | FBG ≥ 125mg/dL or HbA1c ≥ 6.5% or self-reported diagnosis of diabetes, use of insulin, or oral hypoglycemic agents |
|  | Hypertension | SBP ≥ 130mmHg or DBP ≥ 80mmHg or self-reported diagnosis of hypertension, or use of oral antihypertensive agents |
|  | Hypertriglyceridemia | Triglycerides ≥ 135mg/dL |
|  | Metabolic syndrome | Metabolic syndrome is defined by the presence of 3 or more of the following:  a) WC ≥ 88/102 cm if female/male (or if Asian ancestry ≥ 80/90 cm in female/male)  b) HDL-C < 50/40 mg/dL in female/male  c) Triglycerides ≥ 150mg/dL  d) Elevated blood pressure (SBP ≥130 mmHg, or DBP ≥80 mmHg, and/or antihypertensive use]  e) Prediabetes |

**Abbreviations:** CKM: cardiovascular-kidney-metabolic, CVD: cardiovascular disease, CHF: congestive heart failure, CHD: congestive heart failure, AP: angina/angina pectoris, CKD: chronic kidney disease, KDIGO: The Kidney Disease: Improving Global Outcomes, eGFR: estimated glomerular filtration rate, UACR: urinary albumin to creatinine ratio, BMI: body mass index, WC: waist circumference, FBG: fasting blood glucose, HbA1c: glycohemoglobin, SBP: systolic blood pressure, DBP: diastolic blood pressure, HDL-C: high-density lipoprotein cholesterol.

**Table S2** Detailed algorithm of the simplified 10-year CVD risk models

| Male | Log-odds = -3.031168+0.7688528*(age-55)/10+0.0736174*(TC-HDL-C-3.5)-0.0954431*(HDL-C-1.3)/0.3-0.4347345*(min(SBP, 110)-110)/20+0.3362658*(max(SBP,110)-130) /20+0.7692857*(if diabetes)+0.4386871*(if current smoker) +0.5378979*(min(eGFR,60)-60)/-15+0.0164827*(max(eGFR,60)-90)/-15+0.288879*(if using antihypertensive agents)-0.1337349*(if using statin)-0.0475924*(if using antihypertensive agents)*(max(SBP,110)-130)/20+0.150273*(if using statin)*(TC-HDL-C-3.5)-0.0517874*(age-55)/10*(TC-HDL-C-3.5)+0.0191169*(age-55)/10*(HDL-C-1.3)/0.3-0.1049477*(age-55)/10*(max(SBP,110)-130)/20-0.2251948*(age-55)/10*(if diabetes)-0.0895067*(age-55)/10*(if current smoker)-0.1543702*(age-55)/10*(min(eGFR,60)-60)/-15  Risk = exp (log-odds)/(1+exp(log-odds)) |
| --- | --- |
| Female | Log-odds = -3.307728+0.7939329*(age-55)/10 +0.0305239*(TC-HDL-C-3.5)-0.1606857*(HDL-C-1.3)/0.3-0.2394003*(min(SBP, 110)-110)/20+0.360078*(max(SBP, 110)-130) /20+0.8667604*(if diabetes)+0.5360739*(if current smoker)+0.6045917*(min(eGFR,60)-60)/-15+0.0433769*(max(eGFR, 60)-90)/-15+0.3151672*(if using antihypertensive agents)-0.1477655*(if using statin)-0.0663612*(if using antihypertensive agents)*(max(SBP, 110)-130)/20+0.1197879*(if using statin)*(TC-HDL-C-3.5)-0.0819715*(age-55)/10*(TC-HDL-C-3.5)+0.0306769*(age-55)/10*(HDL-C-1.3)/0.3-0.0946348*(age-55)/10*(max(SBP, 110)-130)/20-0.27057*(age-55)/10*(if diabetes)-0.078715*(age-55) /10*(if current smoker)-0.1637806*(age-55)/10*(min(eGFR,60)-60)/-15  Risk = exp (log-odds)/(1+exp(log-odds)) |

**Abbreviations:** eGFR: estimated glomerular filtration rate, SBP: systolic blood pressure, TC: total cholesterol, HDL-C: high-density lipoprotein cholesterol.

**Table S3** Assessment of CKM Syndrome Stages

| **Stage 0** | Individuals meet all following criteria:  a) BMI < 25 kg/m^2^ (if Asian ancestry < 23 kg/m^2^)  b) WC < 88/102 cm if female/male (if Asian ancestry < 80/90 cm in female/male)  c) Without metabolic risk factors |
| --- | --- |
| **Stage 1** | Individuals meet any of the following criteria:  a) BMI ≥ 25 kg/m^2^ (or ≥ 23 kg/m^2^ if Asian ancestry)  b) WC ≥ 88/102 cm if female/male (if Asian ancestry ≥ 80/90 cm in female/male)  c) Prediabetes |
| **Stage 2** | Individuals meet any of the following criteria:  a) Individuals with moderate-to-high-risk CKD (per KDIGO criteria)  b) Metabolic risk factors |
| **Stage 3** | Individuals meet any of the following criteria:  a) Very-high-risk CKD (per KDIGO criteria)  b) High-predicted 10-year cardiovascular risk (≥20%), estimated using the AHA PREVENT equations: |
| **Stage 4** | Individuals with clinical CVD. |

**Abbreviations:** CKM: cardiovascular-kidney-metabolic, CVD: cardiovascular disease, CKD: chronic kidney disease, KDIGO: The Kidney Disease: Improving Global Outcomes, BMI: body mass index, WC: waist circumference.

**Table S4.** The collinearity screening of baseline characteristics

| Characteristics | Variance inflation factor |
| --- | --- |
| Age | 2.9 |
| Sex | 1.6 |
| Race | 1.2 |
| Education level | 1.5 |
| Marital status | 1.1 |
| PIR | 1.4 |
| Smoking status | 1.1 |
| Diabetes mellitus | 3.8 |
| Hypertension | 3.2 |
| Alcohol user | 1.1 |
| BMI | 1.4 |
| TG | 1.3 |
| TC | NA |
| HDL-C | 1.5 |
| LDL-C | 1.1 |
| FBG | 3.2 |
| HbA1c | 3.6 |
| ALT | 1.9 |
| AST | 1.7 |
| Uric acid | 1.7 |
| Blood urea nitrogen | 1.6 |
| eGFR | 3 |
| Hypoglycemic agent | 3.2 |
| Hypotensive agent | 3.5 |
| Lipid-lowing agent | 1.4 |
| AGR | 1.2 |

**Abbreviations:** BMI, body mass index; PIR, poverty-to-income rations; TG, triglyceride; TC, total cholesterol; HDL-C, high density lipoprotein cholesterol; LDL-C, low density lipoprotein cholesterol; FBG, fasting blood glucose; HbA1c, glycohemoglobin; eGFR, estimated glomerular filtration rate; ALT, alanine aminotransferase; AST, aspartate aminotransferase.

We think collinearity exists and eliminate these covariables in the final models if their variance inflation factors are greater than or equal to 5.

**Table S5.** Associations of covariates with mortality (N = 15895)

| Covariates | exp(beta) | 95%CI | P Value |
| --- | --- | --- | --- |
| Age | 1.1027 | 1.0977-1.1078 | < 0.0001 |
| Female | 0.6576 | 0.5946-0.7273 | < 0.0001 |
| Race |  |  |  |
| Mexican American | reference | reference | reference |
| Non-Hispanic White | 0.7627 | 0.5814-1.0005 | 0.0504 |
| Non-Hispanic Black | 2.1898 | 1.8632-2.5735 | < 0.0001 |
| Other Race | 0.5581 | 0.4180-0.7451 | 0.0001 |
| Education level |  |  |  |
| Less than 9th grade | reference | reference | reference |
| 9-11th grade | 0.7331 | 0.6188-0.8686 | 0.0003 |
| High school graduate | 0.6386 | 0.5461-0.7468 | < 0.0001 |
| College degree | 0.4051 | 0.3451-0.4754 | < 0.0001 |
| College and above | 0.3316 | 0.2781-0.3954 | < 0.0001 |
| Marital status |  |  |  |
| Married/living with partner | reference | reference | reference |
| Widowed/divorced/separated | 2.5608 | 2.3000-2.8512 | < 0.0001 |
| Never married | 0.4365 | 0.3594-0.5302 | < 0.0001 |
| PIR | 0.8734 | 0.8450-0.9028 | < 0.0001 |
| Smoking status |  |  |  |
| Not at all | reference | reference | reference |
| Some day | 2.4848 | 2.2184-2.7833 | < 0.0001 |
| Every day | 1.5197 | 1.3303-1.7360 | < 0.0001 |
| Diabetes mellitus | 2.8860 | 2.5616-3.2515 | < 0.0001 |
| Hypertension | 3.4812 | 3.1414-3.8578 | < 0.0001 |
| Alcohol user | 0.6169 | 0.5427-0.7013 | < 0.0001 |
| BMI | 0.9834 | 0.9756-0.9913 | < 0.0001 |
| TG | 1.1204 | 1.0832-1.1590 | < 0.0001 |
| HDL-C | 0.9955 | 0.8837-1.1215 | 0.9415 |
| LDL-C | 0.8499 | 0.8030-0.8995 | < 0.0001 |
| FBG | 1.0079 | 1.0068-1.0089 | < 0.0001 |
| HbA1c | 1.3064 | 1.2615-1.3529 | < 0.0001 |
| ALT | 0.9887 | 0.9843-0.9931 | < 0.0001 |
| AST | 1.0129 | 1.0080-1.0177 | < 0.0001 |
| Uric acid | 1.2719 | 1.2302-1.3150 | < 0.0001 |
| Blood urea nitrogen | 1.1006 | 1.0921-1.1091 | < 0.0001 |
| eGFR | 0.9537 | 0.9514-0.9559 | < 0.0001 |
| Hypoglycemic agent | 0.4023 | 0.3521-0.4596 | < 0.0001 |
| Hypotensive agent | 3.7841 | 3.4177-4.1898 | < 0.0001 |
| Lipid-lowing agent | 2.4620 | 2.2063-2.7472 | < 0.0001 |

**Abbreviations:** BMI, body mass index; PIR, poverty-to-income rations; TG, triglyceride; HDL-C, high density lipoprotein cholesterol; LDL-C, low density lipoprotein cholesterol; FBG, fasting blood glucose; HbA1c, glycohemoglobin; eGFR, estimated glomerular filtration rate; ALT, alanine aminotransferase; AST, aspartate aminotransferase.

**Table S6.** The adjusting roles of potential confounders on the estimates of AGR on mortality

| +/- covariates | Basic model | Complete model | The selected covariates |
| --- | --- | --- | --- |
| Initial regression coefficient of AGR | -0.9881 | -0.8746 |  |
| Age | -0.9878 | -0.9865* | Yes |
| Sex | -1.2320* | -0.8136 | Yes |
| Race | -1.0990* | -0.7709* | Yes |
| Education level | -0.8196* | -0.9145 | Yes |
| Marital status | -0.8718* | -0.9020 | Yes |
| PIR | -0.8980 | -0.9066 |  |
| Smoking status | -1.0827 | -0.9111 |  |
| Diabetes mellitus | -0.8492* | -0.8795 | Yes |
| Hypertension | -0.8193* | -0.8773 | Yes |
| Alcohol user | -1.0076 | -0.8800 |  |
| BMI | -1.1543* | -0.8295 |  |
| TG | -0.9426 | -0.8128 |  |
| HDL-C | -0.9885 | -0.8666 |  |
| LDL-C | -0.9964 | -0.8756 |  |
| FBG | -0.8701* | -0.8682 | Yes |
| HbA1c | -0.8129* | -0.8640 | Yes |
| ALT | -0.9621 | -0.8792 |  |
| AST | -0.9837 | -0.9074 |  |
| Uric acid | -0.9637 | -0.8572 |  |
| Blood urea nitrogen | -1.0332 | -0.8724 |  |
| eGFR | -0.9594 | -0.8764 |  |
| Hypoglycemic agent | -0.9119 | -0.8830 |  |
| Hypotensive agent | -0.9276 | -0.8739 |  |
| Lipid-lowing agent | -1.0424 | -0.9260 |  |

*These confounders changed the estimates of AGR on mortality by more than 10% when introduce covariates into the basic model or remove covariates from the complete model

**Table S7.** The selected covariates

| Y | X | The selected covariates (Criterion 1) | The selected covariates (Criterion 2) |
| --- | --- | --- | --- |
| Mortality | AGR | Age, Sex, Race, Education level, Marital status, Diabetes mellitus, Hypertension, FBG, HbA1c | Age, Sex, Race, Education level, Marital status, Diabetes mellitus, Hypertension, FBG, HbA1c |

Criterion 1: These confounders changed the estimates of AGR on mortality by more than 10% when introduce covariates into the basic model or remove covariates from the complete model (Table S6).

Criterion 2: These variables were significantly associated with mortality (P < 0.10) or changed the estimates of AGR on mortality by more than 10% (Table S5 + Table S6)

**Table S8** STROBE Statement—Checklist of items that should be included in reports of cohort studies

|  | Item No | Recommendation | Page No |
| --- | --- | --- | --- |
| **Title and abstract** | 1 | (*a*) Indicate the study’s design with a commonly used term in the title or the abstract | 2 |
|  |  | (*b*) Provide in the abstract an informative and balanced summary of what was done and what was found | 2-3 |
| Introduction | | |  |
| Background/rationale | 2 | Explain the scientific background and rationale for the investigation being reported | 4-5 |
| Objectives | 3 | State specific objectives, including any prespecified hypotheses | 5 |
| Methods | | |  |
| Study design | 4 | Present key elements of study design early in the paper | 5-6 |
| Setting | 5 | Describe the setting, locations, and relevant dates, including periods of recruitment, exposure, follow-up, and data collection | 6 |
| Participants | 6 | (*a*) Give the eligibility criteria, and the sources and methods of selection of participants. Describe methods of follow-up | 6, 10 |
|  |  | (*b*) For matched studies, give matching criteria and number of exposed and unexposed | - |
| Variables | 7 | Clearly define all outcomes, exposures, predictors, potential confounders, and effect modifiers. Give diagnostic criteria, if applicable | 6-10 |
| Data sources/ measurement | 8* | For each variable of interest, give sources of data and details of methods of assessment (measurement). Describe comparability of assessment methods if there is more than one group | 6-7 |
| Bias | 9 | Describe any efforts to address potential sources of bias | 11 |
| Study size | 10 | Explain how the study size was arrived at | 6 |
| Quantitative variables | 11 | Explain how quantitative variables were handled in the analyses. If applicable, describe which groupings were chosen and why | 10 |
| Statistical methods | 12 | (*a*) Describe all statistical methods, including those used to control for confounding | 10-12 |
|  |  | (*b*) Describe any methods used to examine subgroups and interactions | 11 |
|  |  | (*c*) Explain how missing data were addressed | 12 |
|  |  | (*d*) If applicable, explain how loss to follow-up was addressed | 6 |
|  |  | (*e*) Describe any sensitivity analyses | 11-12 |
| Results | | |  |
| Participants | 13* | (*a*) Report numbers of individuals at each stage of study—eg numbers potentially eligible, examined for eligibility, confirmed eligible, included in the study, completing follow-up, and analysed | 6 |
|  |  | (*b*) Give reasons for non-participation at each stage | 6 |
|  |  | (*c*) Consider use of a flow diagram | 6 |
| Descriptive data | 14* | (*a*) Give characteristics of study participants (eg demographic, clinical, social) and information on exposures and potential confounders | 12-13 |
|  |  | (*b*) Indicate number of participants with missing data for each variable of interest | 6 |
|  |  | (*c*) Summarise follow-up time (eg, average and total amount) | 2 |
| Outcome data | 15* | Report numbers of outcome events or summary measures over time | 2 |
| Main results | 16 | (*a*) Give unadjusted estimates and, if applicable, confounder-adjusted estimates and their precision (eg, 95% confidence interval). Make clear which confounders were adjusted for and why they were included | 10-11, 13 |
|  |  | (*b*) Report category boundaries when continuous variables were categorized | 11 |
|  |  | (*c*) If relevant, consider translating estimates of relative risk into absolute risk for a meaningful time period | - |
| Other analyses | 17 | Report other analyses done—eg analyses of subgroups and interactions, and sensitivity analyses | 13-15 |
| Discussion | | |  |
| Key results | 18 | Summarise key results with reference to study objectives | 15 |
| Limitations | 19 | Discuss limitations of the study, taking into account sources of potential bias or imprecision. Discuss both direction and magnitude of any potential bias | 21-22 |
| Interpretation | 20 | Give a cautious overall interpretation of results considering objectives, limitations, multiplicity of analyses, results from similar studies, and other relevant evidence | 21 |
| Generalisability | 21 | Discuss the generalisability (external validity) of the study results | 21 |
| Other information | | |  |
| Funding | 22 | Give the source of funding and the role of the funders for the present study and, if applicable, for the original study on which the present article is based | 23 |

* Give information separately for exposed and unexposed groups.

**Table S9** Weighted baseline characteristics of included and excluded participants in NHANES 2003-2018

|  | Included (n = 15895) | Excluded (n = 64417) | P-value |
| --- | --- | --- | --- |
| Age (year) | 47.20 ± 0.25 | 33.40 ± 0.20 | < 0.0001 |
| **Sex, % (SE)** |  |  | 0.5282 |
| Male | 49.14 (0.48) | 48.80 (0.23) |  |
| Female | 50.86 (0.48) | 51.20 (0.23) |  |
| **Race, % (SE)** |  |  | < 0.0001 |
| Mexican American | 8.47 (0.64) | 10.77 (0.74) |  |
| Non-Hispanic White | 68.82 (1.18) | 62.24 (1.33) |  |
| Non-Hispanic Black | 10.18 (0.61) | 12.86 (0.72) |  |
| Other Race | 12.52 (0.60) | 14.13 (0.56) |  |
| **Education level, % (SE)** |  |  | 0.0006 |
| Less than 9th grade | 5.56 (0.28) | 5.90 (0.24) |  |
| 9-11th grade | 10.48 (0.46) | 10.93 (0.36) |  |
| High school graduate | 23.00 (0.56) | 24.09 (0.50) |  |
| College degree | 31.30 (0.67) | 31.18 (0.42) |  |
| College and above | 29.61 (0.95) | 27.74 (0.79) |  |
| **Marital status, % (SE)** |  |  | < 0.0001 |
| Married/living with partner | 65.46 (0.66) | 59.16 (0.59) |  |
| Widowed/divorced/separated | 17.41 (0.43) | 18.84 (0.33) |  |
| Never married | 17.10 (0.59) | 22.13 (0.54) |  |
| **PIR, % (SE)** |  |  | < 0.0001 |
| < 1.3 | 20.15 (0.68) | 26.24 (0.69) |  |
| 1.3-3.0 | 28.90 (0.68) | 29.62 (0.53) |  |
| ≥ 3.0 | 50.95 (0.99) | 44.14 (0.91) |  |
| **Smoking status, % (SE)** |  |  | < 0.0001 |
| Every day | 20.68 (0.56) | 13.48 (0.27) |  |
| Some day | 25.31 (0.59) | 15.16 (0.28) |  |
| Not at all | 54.01 (0.71) | 71.36 (0.39) |  |
| **Diabetes mellitus, % (SE)** |  |  | < 0.0001 |
| Yes | 9.10 (0.29) | 6.20 (0.17) |  |
| No | 90.90 (0.29) | 93.80 (0.17) |  |
| **Hypertension, % (SE)** |  |  | < 0.0001 |
| Yes | 31.24 (0.61) | 28.59 (0.43) |  |
| No | 68.76 (0.61) | 71.41 (0.43) |  |
| **Alcohol user, % (SE)** |  |  | 0.2993 |
| Yes | 16.08 (0.49) | 15.56 (0.40) |  |
| No | 83.92 (0.49) | 84.44 (0.40) |  |
| **BMI (kg/m2)** | 28.74 ± 0.09 | 26.06 ± 0.07 | < 0.0001 |
| **Laboratory parameters** |  |  |  |
| Leukocyte (1000 cell/μL) | 6.79 ± 0.03 | 7.53 ± 0.02 | < 0.0001 |
| TG (mmol/L) | 1.44 ± 0.01 | 1.25 ± 0.02 | < 0.0001 |
| TC (mmol/L) | 5.00 ± 0.01 | 4.53 ± 0.02 | < 0.0001 |
| HDL-C (mmol/L) | 1.41 ± 0.01 | 1.37 ± 0.01 | < 0.0001 |
| LDL-C (mmol/L) | 2.96 ± 0.01 | 2.61 ± 0.01 | < 0.0001 |
| FBG (mg/dL) | 105.36 ± 0.33 | 103.08 ± 0.46 | < 0.0001 |
| HbA1c (%) | 5.58 ± 0.01 | 5.54 ± 0.01 | < 0.0001 |
| ALT (U/L) | 24.10 ± 0.13 | 23.11 ± 0.10 | < 0.0001 |
| AST (U/L) | 24.24 ± 0.11 | 24.42 ± 0.08 | 0.1454 |
| Albumin (g/dL) | 4.23 ± 0.01 | 4.30 ± 0.01 | < 0.0001 |
| Globulin (g/dL) | 2.86 ± 0.01 | 2.83 ± 0.01 | < 0.0001 |
| Uric acid (mg/dL) | 5.44 ± 0.02 | 5.32 ± 0.01 | < 0.0001 |
| Blood urea nitrogen (mg/dL) | 13.29 ± 0.07 | 13.02 ± 0.06 | < 0.0001 |
| eGFR (ml/min/1.73m^2^) | 98.56 ± 0.34 | 97.65 ± 0.48 | 0.0006 |
| **Drug use** |  |  |  |
| **Hypoglycemic agent, % (SE)** |  |  | < 0.0001 |
| Yes | 49.80 (1.50) | 40.18 (0.95) |  |
| No | 50.20 (1.50) | 59.82 (0.95) |  |
| **Hypotensive agent, % (SE)** |  |  | 0.2990 |
| Yes | 86.84 (0.87) | 85.55 (0.57) |  |
| No | 13.16 (0.87) | 14.45 (0.57) |  |
| **Lipid-lowing agent, % (SE)** |  |  | 0.2691 |
| Yes | 77.86 (1.17) | 76.69 (0.84) |  |
| No | 22.14 (1.17) | 23.31 (0.84) |  |
| **Outcomes, % (SE)** |  |  |  |
| All-cause mortality | 7.85 (0.34) | 9.73 (0.28) | < 0.0001 |
| Cardiovascular mortality | 2.19 (0.14) | 1.95 (0.09) | 0.0826 |

Values are presented as the mean ± standard error (SE) unless stated otherwise.

**Abbreviations:** BMI, body mass index; PIR, poverty-to-income rations; TG, triglyceride; TC, total cholesterol; HDL-C, high density lipoprotein cholesterol; LDL-C, low density lipoprotein cholesterol; FBG, fasting blood glucose; HbA1c, glycohemoglobin; eGFR, estimated glomerular filtration rate; ALT, alanine aminotransferase; AST, aspartate aminotransferase.

**Table S10** HR (95%CI) for all-cause and CVD mortality according to AGR among participants with CKM syndrome in NHANES 2003-2018 (**following-up > 2 years**)

|  | AGR | | | | |  |
| --- | --- | --- | --- | --- | --- | --- |
|  | Q1 (0.44-1.26) | Q2 (1.27-1.44) | Q3 (1.45-1.63) | Q4 (1.64-5.88) | P trend | AGR continuous |
| **All-cause mortality** |  |  |  |  |  |  |
| Number of deaths (%) | 485 (13.94) | 385 (10.69) | 324 (8.59) | 287 (7.51) |  | 1481 (10.09) |
| Model 1 | 1.00 | 0.68 (0.57, 0.80) | 0.53 (0.42, 0.65) | 0.50 (0.42, 0.59) | < 0.0001 | 0.34 (0.26, 0.46) |
| P-value |  | < 0.0001 | < 0.0001 | < 0.0001 |  | < 0.0001 |
| Model 2 | 1.00 | 0.66 (0.54, 0.80) | 0.51 (0.41, 0.64) | 0.52 (0.42, 0.64) | < 0.0001 | 0.36 (0.26, 0.50) |
| P-value |  | < 0.0001 | < 0.0001 | < 0.0001 |  | < 0.0001 |
| Model 3 | 1.00 | 0.67 (0.55, 0.81) | 0.56 (0.45, 0.70) | 0.58 (0.47, 0.71) | < 0.0001 | 0.38 (0.25, 0.57) |
| P-value |  | < 0.0001 | < 0.0001 | < 0.0001 |  | < 0.0001 |
| **CVD mortality** |  |  |  |  |  |  |
| Number of deaths (%) | 150 (4.31) | 127 (3.52) | 104 (2.76) | 80 (2.09) |  | 461 (3.14) |
| Model 1 | 1.00 | 0.71 (0.52, 0.97) | 0.49 (0.35, 0.67) | 0.39 (0.30, 0.51) | < 0.0001 | 0.24 (0.15, 0.38) |
| P-value |  | 0.0303 | < 0.0001 | < 0.0001 |  | < 0.0001 |
| Model 2 | 1.00 | 0.72 (0.51, 1.02) | 0.46 (0.32, 0.66) | 0.42 (0.30, 0.60) | < 0.0001 | 0.24 (0.13, 0.43) |
| P-value |  | 0.0628 | < 0.0001 | < 0.0001 |  | < 0.0001 |
| Model 3 | 1.00 | 0.73 (0.52, 1.04) | 0.49 (0.33, 0.73) | 0.47 (0.34, 0.67) | < 0.0001 | 0.27 (0.15, 0.48) |
| P-value |  | 0.0783 | 0.0004 | < 0.0001 |  | < 0.0001 |

Model 1: Non-adjusted; Model 2: Adjusted for age, sex and race; Model 3: Adjusted for age, sex, race, education level, marital status, diabetes mellitus, hypertension, FBG, and HbA1c.

**Table S11** HR (95%CI) for all-cause and CVD mortality according to AGR among participants with CKM syndrome in NHANES 2003-2018 (**without cancer and CVD at baseline**)

|  | AGR | | | | |  |
| --- | --- | --- | --- | --- | --- | --- |
|  | Q1 (0.40-1.26) | Q2 (1.27-1.44) | Q3 (1.45-1.63) | Q4 (1.64-5.88) | P trend | AGR continuous |
| **All-cause mortality** |  |  |  |  |  |  |
| Number of deaths (%) | 542 (14.03) | 407 (10.54) | 340 (8.63) | 300 (7.70) |  | 1593 (10.20) |
| Model 1 | 1.00 | 0.69 (0.56, 0.85) | 0.50 (0.40, 0.63) | 0.43 (0.34, 0.55) | < 0.0001 | 0.27 (0.19, 0.39) |
| P-value |  | 0.0006 | < 0.0001 | < 0.0001 |  | < 0.0001 |
| Model 2 | 1.00 | 0.69 (0.55, 0.88) | 0.49 (0.38, 0.64) | 0.47 (0.35, 0.62) | < 0.0001 | 0.29 (0.19, 0.43) |
| P-value |  | 0.0021 | < 0.0001 | < 0.0001 |  | < 0.0001 |
| Model 3 | 1.00 | 0.66 (0.52, 0.85) | 0.52 (0.40, 0.67) | 0.50 (0.37, 0.67) | < 0.0001 | 0.32 (0.21, 0.49) |
| P-value |  | 0.0011 | < 0.0001 | < 0.0001 |  | < 0.0001 |
| **CVD mortality** |  |  |  |  |  |  |
| Number of deaths (%) | 163 (4.22) | 135 (3.50) | 112 (2.84) | 76 (1.95) |  | 486 (3.11) |
| Model 1 | 1.00 | 0.61 (0.39, 0.94) | 0.48 (0.32, 0.72) | 0.30 (0.19, 0.46) | < 0.0001 | 0.17 (0.09, 0.34) |
| P-value |  | 0.0261 | 0.0004 | < 0.0001 |  | < 0.0001 |
| Model 2 | 1.00 | 0.71 (0.44, 1.13) | 0.51 (0.32, 0.82) | 0.37 (0.22, 0.63) | 0.0007 | 0.21 (0.09, 0.48) |
| P-value |  | 0.1460 | 0.0055 | 0.0002 |  | 0.0002 |
| Model 3 | 1.00 | 0.66 (0.41, 1.08) | 0.52 (0.32, 0.84) | 0.39 (0.23, 0.66) | 0.0032 | 0.22 (0.09, 0.51) |
| P-value |  | 0.0984 | 0.0074 | 0.0004 |  | 0.0004 |

Model 1: Non-adjusted; Model 2: Adjusted for age, sex and race; Model 3: Adjusted for age, sex, race, education level, marital status, diabetes mellitus, hypertension, FBG, and HbA1c.

**Table S12** HR (95%CI) for all-cause and CVD mortality according to AGR among participants with CKM syndrome in NHANES 2003-2018 (**without drug use at baseline**)

|  | AGR | | | | |  |
| --- | --- | --- | --- | --- | --- | --- |
|  | Q1 (0.40-1.26) | Q2 (1.27-1.44) | Q3 (1.45-1.63) | Q4 (1.64-5.88) | P trend | AGR continuous |
| **All-cause mortality** |  |  |  |  |  |  |
| Number of deaths (%) | 205 (8.51) | 133 (5.27) | 146 (5.38) | 114 (4.10) |  | 598 (5.73) |
| Model 1 | 1.00 | 0.54 (0.40, 0.73) | 0.56 (0.43, 0.73) | 0.40 (0.29, 0.56) | < 0.0001 | 0.30 (0.18, 0.49) |
| P-value |  | < 0.0001 | < 0.0001 | < 0.0001 |  | < 0.0001 |
| Model 2 | 1.00 | 0.51 (0.37, 0.69) | 0.50 (0.38, 0.67) | 0.37 (0.26, 0.52) | < 0.0001 | 0.28 (0.17, 0.46) |
| P-value |  | < 0.0001 | < 0.0001 | < 0.0001 |  | < 0.0001 |
| Model 3 | 1.00 | 0.49 (0.19, 1.24) | 0.56 (0.22, 1.44) | 0.34 (0.12, 0.95) | < 0.0001 | 0.17 (0.05, 0.63) |
| P-value |  | 0.1359 | 0.2324 | 0.0409 |  | 0.0081 |
| **CVD mortality** |  |  |  |  |  |  |
| Number of deaths (%) | 46 (1.91) | 36 (1.43) | 43 (1.59) | 27 (0.97) |  | 152 (1.46) |
| Model 1 | 1.00 | 0.64 (0.34, 1.18) | 0.63 (0.37, 1.09) | 0.37 (0.22, 0.64) | 0.0063 | 0.29 (0.14, 0.63) |
| P-value |  | 0.1530 | 0.1020 | 0.0003 |  | 0.0016 |
| Model 2 | 1.00 | 0.61 (0.32, 1.16) | 0.65 (0.38, 1.11) | 0.39 (0.21, 0.70) | 0.0085 | 0.34 (0.15, 0.77) |
| P-value |  | 0.1344 | 0.1122 | 0.0017 |  | 0.0104 |
| Model 3 | 1.00 | 0.59 (0.31, 1.15) | 0.65 (0.35, 1.21) | 0.42 (0.23, 0.77) | 0.0380 | 0.38 (0.15, 0.92) |
| P-value |  | 0.1245 | 0.1818 | 0.0055 |  | 0.0323 |

Model 1: Non-adjusted; Model 2: Adjusted for age, sex and race; Model 3: Adjusted for age, sex, race, education level, marital status, diabetes mellitus, hypertension, FBG, and HbA1c.

**Table S13** HR (95%CI) for all-cause and CVD mortality according to AGR among participants with CKM syndrome in NHANES 2003-2018 (**Stage 3-4**)

|  | AGR | | | | |  |
| --- | --- | --- | --- | --- | --- | --- |
|  | Q1 (0.44-1.26) | Q2 (1.27-1.44) | Q3 (1.45-1.63) | Q4 (1.64-2.89) | P trend | AGR continuous |
| **All-cause mortality** |  |  |  |  |  |  |
| Number of deaths (%) | 394 (42.97) | 286 (37.58) | 232 (34.63) | 217 (37.22) |  | 1129 (38.52) |
| Model 1 | 1.00 | 0.79 (0.64, 0.98) | 0.67 (0.51, 0.87) | 0.74 (0.58, 0.93) | < 0.0001 | 0.56 (0.41, 0.77) |
| P-value |  | 0.0362 | 0.0026 | 0.0111 |  | 0.0003 |
| Model 2 | 1.00 | 0.74 (0.59, 0.92) | 0.59 (0.45, 0.77) | 0.65 (0.53, 0.81) | < 0.0001 | 0.43 (0.31, 0.60) |
| P-value |  | 0.0066 | 0.0001 | < 0.0001 |  | < 0.0001 |
| Model 3 | 1.00 | 0.74 (0.60, 0.91) | 0.63 (0.48, 0.82) | 0.69 (0.57, 0.86) | < 0.0001 | 0.46 (0.34, 0.64) |
| P-value |  | 0.0052 | 0.0008 | 0.0006 |  | < 0.0001 |
| **CVD mortality** |  |  |  |  |  |  |
| Number of deaths (%) | 136 (14.83) | 107 (14.06) | 78 (11.64) | 73 (12.52) |  | 394 (13.44) |
| Model 1 | 1.00 | 0.89 (0.63, 1.26) | 0.58 (0.38, 0.88) | 0.71 (0.51, 0.97) | 0.0255 | 0.50 (0.30, 0.83) |
| P-value |  | 0.5301 | 0.0103 | 0.0366 |  | 0.0077 |
| Model 2 | 1.00 | 0.83 (0.57, 1.22) | 0.49 (0.31, 0.76) | 0.59 (0.43, 0.83) | 0.0005 | 0.34 (0.19, 0.63) |
| P-value |  | 0.3536 | 0.0013 | 0.0020 |  | 0.0005 |
| Model 3 | 1.00 | 0.85 (0.59, 1.23) | 0.51 (0.33, 0.79) | 0.65 (0.46, 0.92) | 0.0042 | 0.39 (0.22, 0.71) |
| P-value |  | 0.3901 | 0.0028 | 0.0159 |  | 0.0018 |

Model 1: Non-adjusted; Model 2: Adjusted for age, sex and race; Model 3: Adjusted for age, sex, race, education level, marital status, diabetes mellitus, hypertension, FBG, and HbA1c.
